# Herramientas clínicas en el diagnóstico del desarrollo sexual anómalo: ¿del análisis hormonal al análisis genético?

**DOI:** 10.1515/almed-2021-0074

**Published:** 2021-11-03

**Authors:** Rodolfo A. Rey

**Affiliations:** Centro de Investigaciones Endocrinológicas “Dr. César Bergadá” (CEDIE), CONICET – FEI – División de Endocrinología, Hospital de Niños Ricardo Gutiérrez, C1425EFD Buenos Aires, Argentina; Unidad de Medicina Traslacional, Hospital de Niños Ricardo Gutiérrez, C1425EFD Buenos Aires, Argentina; Departamento de Histología, Universidad de Buenos Aires, Facultad de Medicina, Departamento de Histología, Embriología, Biología Celular y Genética, C1121ABG Buenos Aires, Argentina

**Keywords:** estradiol, FSH, hormona antimülleriana, secuenciación de alto rendimiento, testosterona

En la mayoría de las sociedades en todo el mundo se ha tenido una concepción binaria del sexo. ¿“Niña o niño”? suele ser lo primero que se pregunta sobre un recién nacido. Esta visión profundamente arraigada en la sociedad ha determinado la forma de abordar clínicamente a aquellas personas cuyo sexo genético no se correspondía con el sexo de sus gónadas y/o con el aspecto de sus genitales. No fue hasta 2005, que los expertos de los distintos campos relacionados con estas anomalías, incluidos pediatras, endocrinólogos, cirujanos, genetistas, psicólogos y asociaciones de pacientes, se reunieron por primera vez para valorar desde un punto de vista crítico el abordaje de las anomalías intersexuales desde una perspectiva amplia y se consensuaron unas directrices futuras. En el llamado “Consenso de Chicago” se establecieron las bases para el empleo de una nueva nomenclatura, acuñando el término “desarrollo sexual anómalo (DSD)” y, lo que es aún más importante, se trataron aspectos esenciales, como la necesidad de que la investigación y el tratamiento de pacientes con DSD sean llevados a cabo por un equipo multidisciplinar, la recomendación de asignar un género social al recién nacido teniendo en cuenta aspectos culturales de la familia, y la necesidad de realizar estudios prospectivos en los que se analicen los resultados a largo plazo [1]. A pesar del relativamente poco tiempo que ha transcurrido desde entonces, se han producido importantes cambios en el ámbito científico y social, que implican una revisión permanente de este tema [2], [3], [4].

Un aspecto concreto que ha revolucionado la perspectiva adoptada en el diagnóstico de las patologías congénitas es la llegada de las tecnologías de alto rendimiento, tales como la secuenciación de alto rendimiento (NGS). Hasta la primera década del presente siglo, prevaleció la estrategia basada en genes candidatos, en la que se analizan las características anatómicas y hormonales del paciente con DSD [5]. El diagnóstico molecular ofrecía un rendimiento relativamente bajo, excepto para las patologías extremadamente típicas, como el síndrome de insensibilidad completa a los andrógenos (CAIS), el síndrome del conducto mülleriano persistente (PMDS) o la hiperplasia suprerrenal congénita (HSC) (Tabla 1), donde en la mayoría de casos se identificaron las variantes patogénicas (“mutaciones”) en los genes que codifican el receptor de andrógenos, la hormona antimülleriana (AMH) o su receptor, o la enzima 21- hidroxilasa. Por otro lado, mostraba una baja eficiencia diagnóstica en pacientes con disgenesia gonadal, insensibilidad parcial a los andrógenos (PAIS), DSD ovotesticular o DSD con cariotipo 46,XX no HSC. El uso de estos paneles de genes en la clínica, o de la secuenciación del exoma o genoma completo en el contexto de la investigación ha mejorado notablemente el diagnóstico etiológico de los pacientes con DSD [6].

**Tabla 1:** Etiologías genéticas del desarrollo sexual anómalo (DSD).

DSD con cariotipo 46,XY	DSD cromosómicos (45,X/46,XY – 46,XX/46,XY – etc.)	DSD con cariotipo 45,XX
Disgenesia gonadal	Disgenesia gonadal, DSD ovotesticular	DSD testicular, DSD ovotesticular
Anomalías en la síntesis de andrógenos (hipoplasia de células de Leydig, déficit en la esteroidogénesis)		Exceso de andrógenos (HSC, déficit de aromatasa)
Anomalías en la acción de los andrógenos		
Anomalías en la síntesis o acción de AMH (PMDS)		

¿Significa esto el fin de la caracterización clínica de los pacientes con genitales ambiguos o discordancia entre el aspecto de los genitales externos y el cariotipo? El acceso a la NGS es aún limitado. No obstante, aun cuando su uso se haya extendido, una vez se haya reducido su coste, la interpretación de los *big data* generados por la secuenciación del exoma o del genoma dependerá de la validación de la fisiopatología subyacente. Así mismo, uno de los mayores retos de esta tecnología es su capacidad para determinar la patogenicidad de las miles de variantes genéticas detectadas en una lectura, así como garantizar la adherencia a las directrices consensuadas por colegios profesionales como el Colegio Americano de Genética y Genómica Clínica (ACMG) y la Asociación de Patología Molecular [7]. Estas directrices subrayan la importancia de los procedimientos preanalíticos y postanalíticos. De hecho, antes de decidir si realizar una NGS, es esencial realizar una caracterización clínica del paciente; además, cuanto más detallado sea el perfil fenotípico, mayor será el rendimiento diagnóstico de la NGS. Del mismo modo, el hallazgo de variantes de significado incierto (VUS) en genes imprevistos se puede interpretar con mayor facilidad, si se realiza una caracterización precisa del paciente mediante descripciones anatómicas (clínicas, imágenes), junto con análisis hormonales, incluyendo los esteroides sexuales, la AMH y las gonadotropinas [8], [9], [10]. En muchos casos, un diagnóstico genético preciso permitirá un abordaje clínico personalizado. Sin embargo, si no fuera posible realizar un análisis genético sofisticado, realizar una caracterización clínica y endocrinológica resultará de gran utilidad ante un nuevo caso de DSD [8, 9]. De hecho, hay que abordar cada caso de forma individual, basándonos en el cariotipo, la edad de aparición del problema y la apariencia de los genitales internos y externos, así como otros aspectos no reproductivos [10]. Además, la introducción gradual de la espectrometría de masas para la determinación exacta de esteroides en muestras de suero y orina ha mejorado el diagnóstico en los neonatos [11] y los tratamientos posteriores [12], [13], [14].

En conclusión, una caracterización fenotípica exhaustiva mediante la caracterización anatómica y endocrina sigue siendo esencial a la hora de establecer un diagnóstico inicial de DSD con cariotipo 46,XX, 46,XY u otros cariotipos anormales. La ampliación de los paneles de esteroides sexuales para su determinación mediante espectrometría de masas, así como la secuenciación paralela masiva de genes potencialmente implicados, han permitido mejorar la precisión de los diagnósticos etiológicos, mejorando la aplicación de la medicina personalizada en el DSD.
